# Analysis of *in vitro *replicated human hepatitis C virus (HCV) for the determination of genotypes and quasispecies

**DOI:** 10.1186/1743-422X-3-81

**Published:** 2006-09-29

**Authors:** Dennis Revie, Michael O Alberti, Ravi S Braich, Nickolas Chelyapov, David Bayles, John G Prichard, S Zaki Salahuddin

**Affiliations:** 1 Department of Biology, California Lutheran University, Thousand Oaks, California, USA; 2 California Institute of Molecular Medicine, Ventura, California, USA; 3 Ventura County Medical Center, Ventura, California, USA; 4 Alnylam Pharmaceuticals, Cambridge, Massachusetts, USA; 5 University of Southern California, Los Angeles, California, USA

## Abstract

Isolation and self-replication of infectious HCV has been a difficult task. However, this is needed for the purposes of developing rational drugs and for the analysis of the natural virus. Our recent report of an *in vitro *system for the isolation of human HCV from infected patients and their replication in tissue culture addresses this challenge. At California Institute of Molecular Medicine several isolates of HCV, called CIMM-HCV, were grown for over three years in cell culture. This is a report of the analysis of CIMM-HCV isolates for subtypes and quasispecies using a 269 bp segment of the 5'UTR. HCV RNA from three patients and eleven CIMM-HCV were analyzed for this purpose. All isolates were essentially identical. Isolates of HCV from one patient were serially transmitted into fresh cells up to eight times and the progeny viruses from each transmission were compared to each other and also to the primary isolates from the patient's serum. Some isolates were also transmitted to different cell types, while others were cultured continuously without retransmission for over three years. We noted minor sequence changes when HCV was cultured for extended periods of time. HCV in T-cells and non-committed lymphoid cells showed a few differences when compared to isolates obtained from immortalized B-cells. These viruses maintained close similarity despite repeated transmissions and passage of time. There were no subtypes or quasispecies noted in CIMM-HCV.

## Background

HCV infects millions of people throughout the world and is a cause of several serious diseases. It has been estimated that there are over 170 million carriers of HCV worldwide [1]. Until recently, the inability to culture HCV *in vitro *has severely limited meaningful definitive studies leading to therapeutics and vaccines. We have developed a robust *in vitro *system for replicating human HCV and for extended periods of time [2]. Several studies in the past have reported *in vitro *replication of HCV [3-6]. However, none of these have yet demonstrated biologically infectious HCV isolated from patient's blood, or have grown these isolates *in vitro *for a significant amount of time. After our studies were published, others reported culturing synthetic HCV constructs based on Replicon technology. Wakita *et al*. [7] recently reported the development of a full length HCV RNA, JFH-1, that initially needed to be transfected into Huh7 cells. This moiety then could replicate in cell culture and infect other Huh7 cells. Two other studies followed that publication [8,9], and are probably intended as a commercial product for testing therapeutic agents. Bartenschlager and his associates have made a major contribution to the HCV field by developing Replicon technology [10-12]. These Replicon-based systems are non-infectious, and need transfection into the Huh7 cell line or variants thereof. Although a number of studies have been done in non-human primates, the relationship of Replicon systems to human diseases is not known yet. As Huh7 cells are reported to have a defective dsRNA response pathway as well as a defective induction of apoptosis [13], it is likely that the multiplication of Replicons in Huh7 derived cells may be due to the unusual properties of these cells rather than a unique capability of Replicons. Jopling *et al*. [14] suggest that microRNA (mir-122) possibly helps Replicons multiply in Huh7 cells. Su *et al*. [15] have suggested that there is a need for models of HCV infection other than Replicons. We believe that Replicons are not a good system, as the world is not aware of a Replicon-based disease. A meaningful *in vitro *system should isolate infectious viruses from patients that are essentially the same as the entities found in the patients. This meaningful system should also facilitate replication of HCV for a significant amount of time. Although expression of a relatively high titer of progeny virus would be desirable, this should not be a requirement, as most slow viruses grow at a low or very low titer. Finally, the isolated HCV should be capable of infecting new target cells without transfection.

A molecular analysis of California Institute of Molecular Medicine isolated HCV (CIMM-HCV) for possible existence of subtypes and quasispecies is reported here. For this analysis, we chose to study the 5'UTR, which is used as a standard for this purpose. The analyzed region includes most of the IRES, which may be important for translation.

The 5'UTR is a 341 nucleotide stretch which is highly conserved among the various strains of HCV RNA obtained from patient sera. Analysis of this region has been used to establish major genotypes [16,17]. Using this system, the common genotypes in the U.S. have been designated 1, 2, and 3. Other regions of the HCV genome are also used to distinguish subtypes from each other. HCV strains can differ from each other by as much as 30% of their sequences [18].

**Figure 1:**
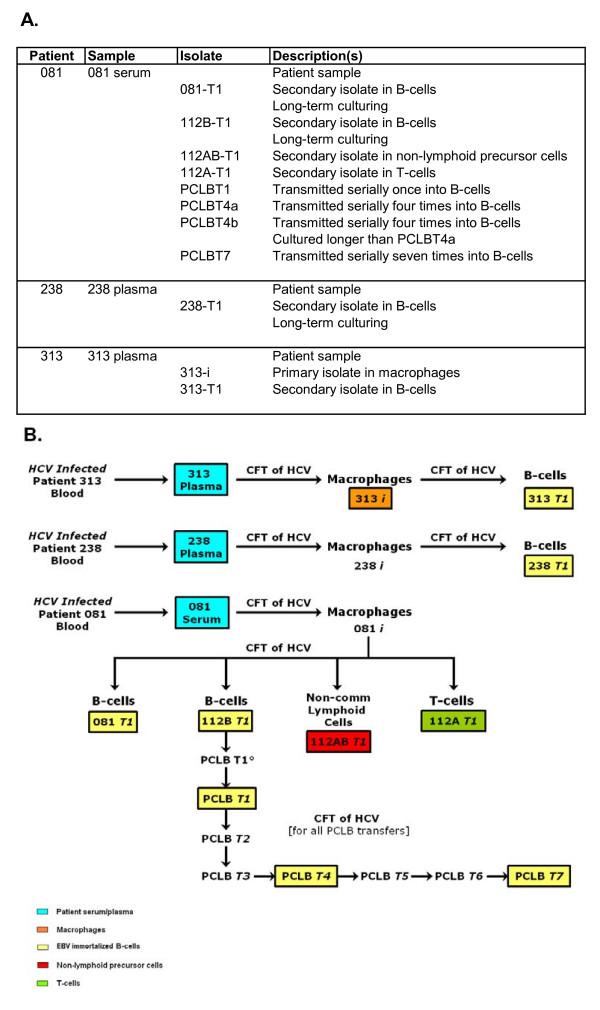
**Isolates used in this study**. A) Listing of isolates and their descriptions. B) Flow chart of isolates. Samples that are in boxes were sequences and analyzed for this report. Cell-free transfers (CFT) of HCV into freshly prepared cells are indicated by arrows. Cell types are indicated by colors.

We have analyzed the 5'UTR of CIMM-HCV and compared them to HCV RNA found in patients' blood. In order to understand *in vitro *produced isolates, we infected different cell types with CIMM-HCV and cultured them for extended periods of time. This was to determine if these transmissions would produce selection favoring additions, deletions, or specific mutations. For the purposes of this report, we have presented data from CIMM-HCV transmitted into macrophages, B-cells, T-cells, and non-committed lymphoid cells. We also compared the progeny of serial transfers into the same cell type over a period of three years. In addition, the CIMM-HCV isolates were also transmitted into hepatocytes and Kuppfer's cells. Extremely low levels of virus were produced by these cells, which prevented meaningful analysis. It is important to note that all analyses presented here relate only to CIMM-HCV (Figure 1A).

## Results

In order to assess whether particular genotypes of HCV were preferentially selected *in vitro*, we analyzed the 5'UTR of HCV RNA representing a number of CIMM-HCV (Figure 1B). We have measured sequence diversity and variation by calculating Shannon entropy and complexity or Pn values [19,20].

### Comparison of the 5'UTR of HCV from patients' blood and CIMM-HCV

**Figure 2:**
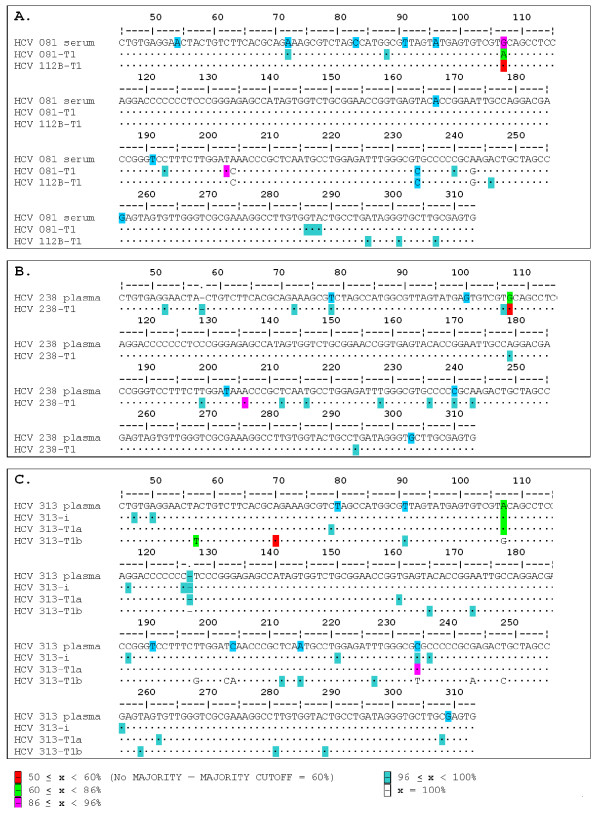
**Comparisons of 5'UTR consensus sequences between patients and isolates**. A) Comparison of patient 081 sera with two HCV isolates: 081-T1, and 112AB-T1. B) Comparison of patient 238 plasma HCV and 238-T1. C) Comparison of patient 313 plasma HCV and three isolates: 313-i, 313-T1a, and 313-T1b.

RNA was purified from patients' sera or plasma and also from CIMM-HCV. In order to determine if these isolates represented the composition of HCV found in patients' sera, sequences were obtained from at least 25 clones for each sample (Table 1). We compared sequences from three patient's sera or plasma and five CIMM-HCV isolates: serum from patient 081 was compared with 081-T1 and 112B-T1, serum from patient 238 was compared with 238-T1, and plasma from 313 was compared with 313-i and 313-T1 (Figure 2). Only one of the primary isolates was analyzed, as these isolates are only a transient stage in the isolation procedure.

**Table 1:** List of CIMM-HCV isolates analyzed^a^

**Isolate**	**No. of clones sequenced**	**Date of transmission**	**Date of HCV isolation and/or RT-PCR**	**Days in culture**
**313 plasma**	60	09/27/2004^b^	10/10/2004	
**313-I**	26	9/27/2004	10/18/2004	21
**313-T1**	26	10/10/2004^c^	10/18/2004	21
				
**238 plasma**	25	08/30/2002^b^	8/30/2002	
**238-T1**	26	09/01/2002^c^	2/24/2005	909
				
**081 serum**	26	03/16/2001^b^	3/16/2001	
**081-T1**	25	04/08/2001^c^	2/24/2005	1441
**112B-T1**	27	04/08/2001^c^	4/24/2005	1500
**112A-T1**	25	04/08/2001^c^	9/11/2001	179
**112AB-T1**	26	04/08/2001^c^	7/21/2001	127
**PCLB-T1**	26	04/08/2001^c^	9/14/2001	182
**PCLB-T4a**	25	08/08/2001^c^	10/12/2001	210
**PCLB-T4b**	26	08/08/2001^c^	2/4/2005	1421
**PCLB-T7**	25	10/02/2001^c^	10/10/2001	208

^a ^Samples from sera and isolates were cloned and sequenced. The dates of transmission indicate the date that HCV was added to the cells. For the T1 through T7 isolates, the exact date of transmission sometimes wasn't known, but the first date of isolation of HCV RNA from that isolate was used to obtain an approximate date.

^b ^Date HCV isolated from blood

^c ^Approximate date of transmission

**Figure 3:**
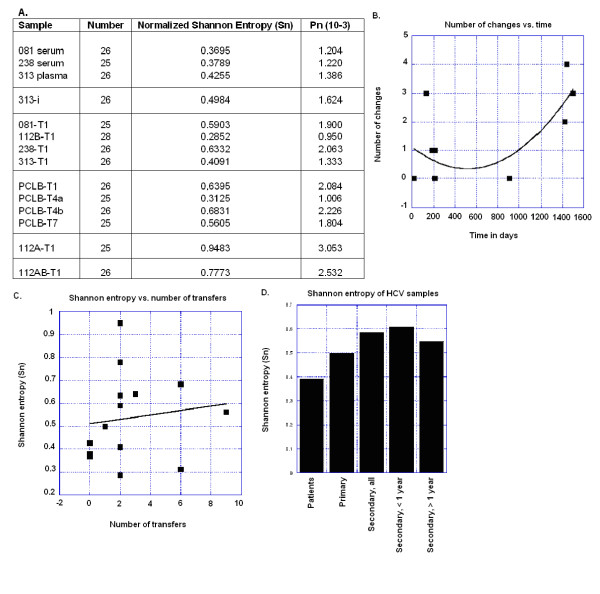
**Variability of CIMM-HCV samples**. A) Sequence complexity of HCV samples. Shannon entropy, normalized for the number of samples and Pn variability as described by Cabot *et al*. (2000) and Pawlotsky *et al*. (1998). B) Number of nucleotide changes in the consensus sequence compared to the consensus of HCV in patient sera. C) Shannon entropy compared against the number of cell-free transfers of HCV into new cell lines. The trend line is a linear fit. D) Comparisons of Shannon entropy against categories of incubation. The error bars represent the standard deviations of the sample entropies. Days of incubation are the days that the isolate was in culture.

Comparisons of HCV from patients 081, 238, and 313 and the corresponding T1 isolates showed that the sequences from 238 and 313 were essentially the same as that of the T1. In two different analyses, the sequences obtained from patient 081 contained 3 and 4 differences compared to the isolates 081-T1 and 112B-T1, respectively. Each isolate had similar distributions of sequences compared to HCV in the patients' blood. The complexity of isolates was higher than the HCV RNA from the blood of the patient (Figure 3A). Isolates 238-T1 and 313-T1 had two common variations in sequences, while 081-T1 had three. HCV present in the sera of patient 313 had large deletions of a part of the 5'UTR. These deletions are described in a separate report [21]. The comparisons of the sera and isolates presented here were performed using only samples containing the entire 5'UTR.

### Distribution of 5'UTR sequences in isolates from patient 081

**Figure 4:**
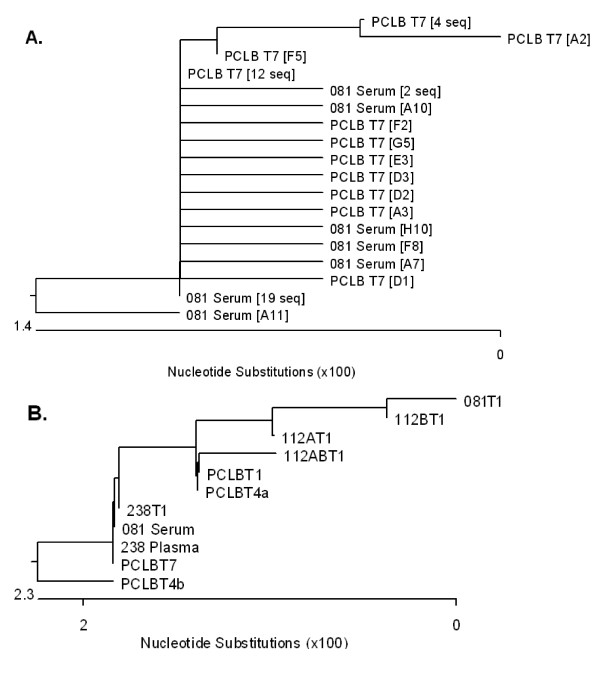
**Comparisons of CIMM-HCV from sera and their corresponding isolates**. The sequences from bases 71 to 315 were aligned using ClustalW. Rooted trees were then drawn using MegAlign in DNASTAR. Branch lengths are proportional to numbers of changes between the sequences. A) Rooted tree of 081 serum and PCLB-T7 sequences. Duplicate sequences from 081 serum and PCLB-T7 were combined into single leaves. Twelve PCLB-T7 sequences were identical to nineteen 081 serum sequences. Most sequences had single base changes compared to these two. B) Rooted tree of 081 and 238 samples.

We compared the sequences from the serum of patient 081 with those found in isolate PCLB-T7 by constructing a rooted neighbor-joining tree (Figure 4A). PCLB-T7 derived from 081 serum which had been transmitted seven times through B-cells (Figure 1B). Twelve sequences of PCLB-T7, and 19 sequences from 081 serum were identical. Five of the sequences from the 081 serum and eight from PCLB-T7 had one change from the consensus, while five of the PCLB-T7 and one of the 081 serum sequences had more than one change as compared to the consensus. We observed minor changes in the distributions of sequences in these samples.

**Figure 5:**
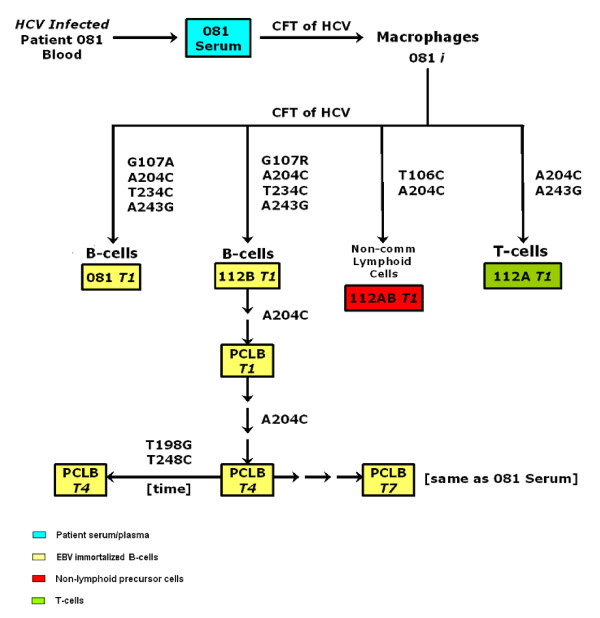
**Consensus changes between 081 serum HCV RNA and corresponding isolates**. The changes shown indicate which bases have changed in the 5'UTR between the patient serum HCV RNA and that isolate. The number is the position changed base, while the first letter is the base in 081 serum and the last base is the base in that isolate. The colors indicate cell types.

Since 081 serum and 238 serum had identical consensus sequences, we constructed another rooted neighbor-joining tree showing the relationship of the various isolates from these two patient samples (Figure 4B). As discussed below, the PCLB-T4b, 112BT1, and 081-T1 samples were cultured for over three years *in vitro*. Changes to the sequence are shown in Figure 5 for each transmission during the extended period of cell culture. There were only minor base changes in these samples.

### Comparison of two isolates from one patient

We isolated HCV on two different occasions from the same patient serum using fresh preparations of transformed B-cells, viz. 081-T1 and 112B-T1 (Figure 2A). Even though both 081-T1 and 112B-T1 had been in culture for over three years, very few changes were seen when compared to each other and to the patient sera. The consensus sequence for the HCV in the patient's blood had a G at position 107, and differed from the two T1 isolates at positions 204 (A vs. C), 234 (T vs. C) and 243 (A vs. G). The only difference in the consensus was at position 107, where 112B had an A or G, while 081 had an A. Both 081-T1 and 112B-T1 had been cultured for almost four years (Table 1). Our data showed few changes in HCV replicated *in vitro *when compared to HCV from patients' sera.

The comparison of 081-T1 and 112B-T1 sequences revealed that each had two common sequences that were exactly the same. Shannon entropy and Pn complexity values showed more variation in the 081-T1 population (Shannon entropy = 0.5903; Pn = 1.900) than the 112B-T1 (Shannon entropy = 0.2852; Pn = 0.950), but the average variation for the two samples was approximately the same as in the patient's sera (Figure 3A).

### Comparison of isolates cultured in different cell types

**Figure 6:**
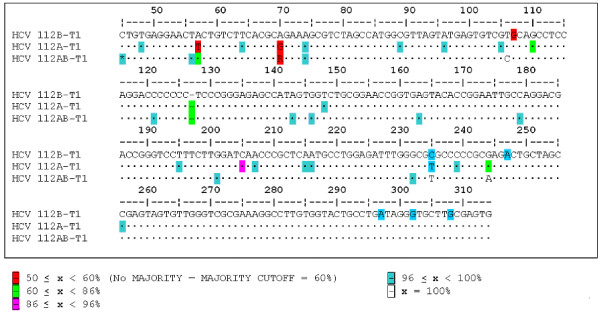
**Comparison of HCV isolates cultured in different cell types**. 112B-T1 was cultured in B-cells, 112A-T1 in T-cells, and 112AB-T1 in non-committed lymphoid cells.

An analysis was performed to determine whether culturing HCV in different cell types would affect the 5'UTR. HCV was transmitted into T-cells (112A-T1) and non-committed lymphoid cells (112AB-T1) (Figure 6). Comparisons of isolates with the 081 serum and the CIMM-HCV from 112B-T1 and 081-T1 showed minor differences. Isolate 112A-T1 differed from 081 serum at two positions (204 and 243). It differed from 112B and 081-T1 at two positions (107 and 234). In addition, 37.5% of the 112A-T1 CIMM-HCV contained an extra C within a C-rich stretch of nucleotides (positions 120 to 126). This extra C was also seen in the 112AB-T1 and 313-T1 samples. Therefore, 112A had minor changes compared to 081 serum, 081-T1, and 112B-T1. 112A-T1 was the only isolate that had no common variant. The Shannon entropy (0.9483) and Pn complexity values (3.053) of 112A were the highest of all CIMM-HCV isolates (Figure 3A).

The 112AB-T1 consensus sequence, compared to 081 serum, had changes at positions 106 and 204. It differed from 081-T1 at three positions (107, 234, and 243). 112A-T1 and 112AB-T1 differed at two positions (106 and 243), while 081-T1 differed from 112AB-T1 at the same two positions and also at position 107. In addition, 27.2% of sequences contained the same extra C within positions 120–126 as did 112A-T1. One significant change for 112AB-T1 was a C in position 106, as all others had a T. Since all of the 112AB-T1 had a C at position 106, there were consistent changes in non-committed lymphoid cells. Although the data suggests that particular types of changes occur when HCV replicates in T-cells and non-committed lymphoid cells, the overall sequence differences compared to HCV from the patient's blood were minor. In summary, the changes in sequences were the same as observed in RNA from patient sera, with the exception of the C in position 106.

### Comparison of isolates after serial transfers *in vitro*

**Figure 7:**
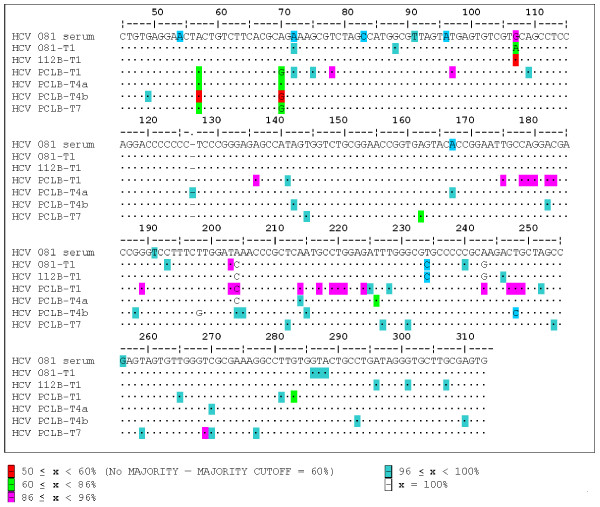
**Comparison of serially transmitted isolates**. HCV from 081 serum was transmitted into 081-T1 and 112B-T1. HCV from 112B-T1 was then serially transmitted seven more times to freshly transformed PCLB cells.

Isolate 112B-T1 was serially transferred seven times into freshly transformed B-cells from human fetal cord blood (PCLB-T1 to PCLB-T7) in order to determine the effects of repeated transfers into a single cell type. We sequenced the 5'UTR of PCLB-T1, PCLB-T4a, and PCLB-T7 and compared these to the 112B-T1 sequence (Figure 7). Each of the transfers into PCLB used fresh cells that were isolated from different human fetal cord blood leukocytes. The comparisons of the consensus sequences showed that 081 serum and PCLB-T1 and PCLB-T4a had one difference at position 204 (A vs. C), while 081 serum and PCLB-T7 had no changes (Figure 4B). Repeated transfers to new cells of the same type resulted in minor variation, but eventually these sequences reverted to that found in the patient sera (Figure 5). The Shannon entropy and Pn complexity numbers for the isolates were higher than HCV found in 081 serum (Figure 3A).

### Impact of long-term *in vitro *cell culture on the fidelity of replication of HCV

We tested the impact of long-term *in vitro *culturing on CIMM-HCV sequences. Samples of PCLB-T4a and PCLB-T4b, which had been cultured for 7 months and 46 months, respectively, were analyzed. We also compared other samples that were cultured for various durations of time. The length of time in culture appeared to have a minor effect on the consensus sequence (Figure 3B). An isolate from patient 238 that was cultured for over 2 years had no changes compared to the sequence of patient HCV RNA (Figure 2B). The two PCLB-T4 HCV samples isolated over three years apart contained changes at positions 198, 204, and 248 (Figure 5). The change at position 204 was a reversion to the sequence found in the patient's sera. For all three of these changes, one of the two isolates had the same base in that position as the patient sample, indicating that the changes were temporary. It was recently reported that patients who were non-responsive to HCV therapy have a G at position 198 [22], which is the same as sample PCLB-T4a. Our isolates had a C or A at position 204, while other reports have found C, A, or U at the same position [22,23]. Neither of these positions are thought to be base paired in the folded 5'UTR. Converting a U to a C in position 248 would not affect base pairing of the stem between domains IIIc and IIId. The small number of changes in the stable HCV-producing cultures may be meaningful in cases such as position 198, or of little consequence, as in the case of position 248. The variations noted in CIMM-HCV were similar to those found in patient RNA [22,23].

In order to assess how the culture period affects the distribution of HCV sequences, Shannon entropies were analyzed (Figure 3). The T1 isolates showed minor increases in variation as determined by this analysis, particularly for T-cells and non-committed lymphoid cells. With time, the sequence variation appears to revert towards the same value found in the serum RNA. Of the four samples that had been cultured for over two years, the entropy of one was lower while the other three had higher entropies compared to the patient's sample.

Since length of time had very little impact on the 5'UTR of the cultured HCV, we investigated whether culturing in different cell types would affect Shannon entropy. Figure 3C shows a plot of the entropy versus the numbers of transfers into new cells. There was a small increase in entropy with number of transfers, but the entropy increases were not significant, as revealed by an unpaired T-test comparing 0 with 6 or more transfers that gave a p value of 0.30.

Comparing the entropy of isolated HCV against that of patients' HCV RNA showed that there were small increases (Figure 3D). The entropy of the secondary samples was 0.58 while the entropy of the patient sample was 0.39. In order to see how the entropy varied, we compared specimens of cultured virus for less than one year, over one year, and cultured in cells other than B cells. The samples cultured for over one year showed a little more entropy than the patients (0.54), while those cultured for less than one year had the highest average entropy of 0.61. This indicates that initially there was greater variation in the isolates, but this variation declined.

### Distribution of variant bases in isolated HCV consensus sequences

**Figure 8:**
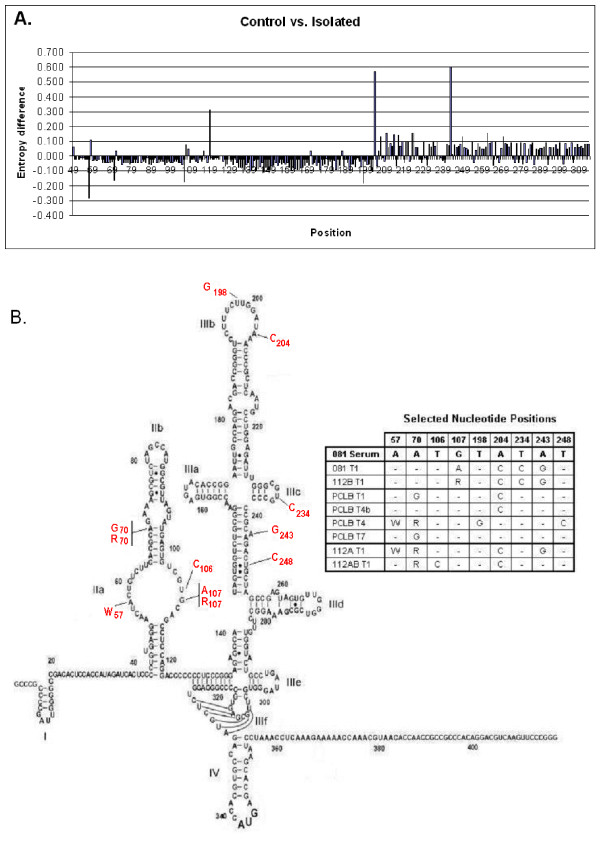
**Variation of CIMM-HCV isolates**. A) Plot of entropy differences between HCV isolated in our system and control sequences. Normalized Shannon entropy values for 5'UTR sequences isolated by our *in vitro *system were subtracted from values determined from control sequences obtained from the HCV sequence database. A negative number is a base that has more variation in the isolates, while a positive number has more variation in the control set of sequences. B) Variation in the bases of 081 HCV isolates. The 081 serum sequence is used for the figure, with changes in the isolates indicated. There were 9 positions where the consensus changed for one or more isolates. The figure is adapted from Lyons *et al*. [26].

In order to determine if the variant bases were located at positions reported by earlier investigators, a control set of sequences obtained from the HCV Sequence Database [24] were compared with sequences from our isolates (Figure 8A). The normalized Shannon entropies of each position of our 190 isolates were compared to 63 sequences of HCV strains 1a and 1b that had been deposited in the HCV Sequence Database. The variation in the isolated samples was greater for positions 57, 106, and 198 than in the control sequences. The primer used to obtain the 5'UTR included base 57. The changes at position 106 were due to the sequences from the non-committed lymphoid cells, all of which contained a C. Position 198 was in a loop. At positions 119, 204, and 243, there was increased variation in the control set of sequences compared to CIMM-HCV. In our samples, positions 204 and 243 had less variation than the control data set. Position 119 is the base adjacent to the string of C's where sometimes an extra C was found, and where the deletion was located in samples from patient 313. The sequences in that region are ACCCCCCCUCCCG, where the A was in position 119. The additions and deletions we are reporting here occur in the C's proximal to A. As shown in Figure 8A, the variation in our isolates was a little greater than the control sequences for bases up to position 203, while the variation in the control sequences were greater for the rest of the 5'UTR.

In order to determine if changes in our isolates were consistent with the current 2D model of the 5'UTR RNA proposed by Honda *et al*. [25], we compared HCV RNA in patient 081 and eight CIMM-HCV from that patient to the existing model. The only variant base that would affect the proposed 2D structure was a C at position 106 in 112AB-T1. The other 22 variant bases were either in regions that are not base paired, or where the changes would not affect base pairing (Figure 8B). The T to C change at position 106 in 112AB-T1 may affect base pairing in the stem of domain II. However, Lyons *et al*. [26] have suggested that position 106 is not in a stem, and therefore base pairing should not be affected.

## Discussion

This study is an analysis of isolates obtained at the California Institute of Molecular Medicine (CIMM). These isolates were studied with respect to the development of subtypes and quasispecies, and also a comparison with HCV RNA found in patient sera. The 5'UTR of HCV RNA was sequenced from eleven CIMM-HCV isolates which were derived from three patients' sera. In two cases, HCV found in the patient sera had the same consensus sequence as our isolates. Although there were minor changes in the isolate from the third patient, the HCV found in the patient was essentially the same despite repeated transfers of those isolates in cell culture. Reports from certified clinical laboratories have suggested that we may have received specimens that included all three major genotypes of HCV present in the U.S. Data reported here indicates that our system produces only one HCV genotype. Comparisons of two isolates from the same patient's blood, 081-T1 and 112B-T1, clearly reflect this phenomenon.

We analyzed at least 25 clones of each sample that had been prepared using two different DNA polymerases, a standard fidelity Taq polymerase and a high fidelity Taq polymerase [27]. The data from these analyses were consistently similar. If changes were caused by the amplification system, we would expect to see variants that would affect base pairing, therefore, polymerases were not a significant player in inducing changes [28,29]. Furthermore, HCV does not seem to produce random mutations as has been noted for HIV-1 [30].

Analysis of CIMM-HCV replicating in different cell types showed minor variations of consensus sequences when compared to the 081 serum HCV. As noted in the results section, we found a C in position 106 for the 112AB-T1, which may affect the formation of a stem-loop in domain II. It is likely that this change would affect the binding of a protein found in lymphoid precursors but not in mature B and T cells.

HCV isolated from T-cells (112A-T1) did not have common sequences, as were seen in our isolates from B-cells. HCV grown in B-cells and non-committed lymphoid cells showed consistent sequence changes, while HCV grown in T-cells had inconsistent changes, therefore lacked sequence commonality. T-cells contained a mixture of T-cell subtypes, including CD4+ and CD8+ cells. Guglietta *et al*. [31] have suggested that CD8+ T-cells help to reduce the HCV population, and the viruses that escape this response have a survival advantage. The survival of the HCV may depend on CD4+ T-cell help [32]. These cells may be responsible for keeping virus production in check in health and imbalanced in disease. These phenomena need further study.

We investigated the 112B-T1 isolate by multiple transfers of progeny virus into freshly transformed B-cells *in vitro *for possible changes in the 5'UTR. Although the consensus sequence of these isolates gradually changed during a series of serial transfers, the last isolate, PCLB-T7, was identical to that of the patient's HCV (081). These changes were small and random. This indicates that we are growing HCV that are indistinguishable from that found in patients' blood.

Culturing HCV for long periods of time could possibly cause genetic changes, therefore we analyzed HCV that had been cultured for over three years. We used two types of experiments: (1) transmission of HCV from patient 081 into different cell types, and (2) transmission of 112B-T1 virus in the same cell type over a period of time. Analysis of the 5'UTR of 081-T1, 112B-T1, and PCLB-T4, which had been cultured for over three years, showed only 3 to 4 base changes. At the same time, isolates from patient 238 that replicated *in vitro *for over two years had no changes in the 5'UTR. This shows the stability of HCV produced in our system.

Comparisons of tissue-dependent differences of HCV have been reported [33-35]. In the case of 081, we saw variability in the same nucleotides that were described by these authors (positions 107, 204, and 243). In addition, we noted variability in base 235. B-cells usually produced HCV with the same consensus sequence as found in the patient, while culturing HCV in other cells types sometimes caused minor changes. It may be that B-cells are the major type of cells that produce HCV found in circulation and may, in turn, infect other cell types. We have previously reported that other cell types also get infected and produce HCV to varying levels and for limited periods of time [2].

As previously reported, we have successfully infected liver cells such as hepatocytes and Kuppfer's cells with CIMM-HCV. However, these cells are short lived and produce HCV at very low titer and for limited times as compared to transformed B-cells. We did not use any commercially available cells, as explained in our previous report. Claims of hepatocytes being the primary target for infection and production of HCV have long been entertained as facts. While we have no problem in accepting the extensive infection of hepatocytes, our view is that the HCV production is probably very low and short-lived. The popular concept, as stated above, may chiefly be inferential due to extensive organ damage, which may result from viral or cellular protein production. The continued replication of HCV in liver cells remains to be convincingly shown, as there are no known long term cultures of either hepatocytes or Kuppfer's cells that can be used to pursue definitive studies. HCV causes liver diseases such as cirrhosis and hepatocellular carcinoma, but this may be due to proteins produced *in situ *in the liver or by cells circulating in the blood. Recently developing information suggests HCV may also cause B-cell lymphoma and possibly other extrahepatic diseases [12,36-38].

Although the macrophages appear to be a necessary first step in our system, their role in HCV replication is unclear. It is important to note that Kuppfer's cells in the liver are essentially macrophages [39] and the hepatocytes are endothelial cells [40]. Macrophages and endothelial cells are distributed throughout the body in various organs systems and vasculature, therefore it is quite possible that HCV infects these cells as well, and are produced by them. However, it is unknown whether the macrophages are modifying or selecting the HCV to make them more infectious. Concentrating the virus or simply removing defective HCV that interfere with further infection of possible target cells are among other possibilities.

It is probable that there is only one type of infectious and replicating HCV. The variety of HCV RNA in patients could result from changes induced in the plasma/sera, including the production of noninfectious viruses. We have drawn these conclusions based our studies of CIMM-HCV only. We also feel that the immune system may not have as significant a role on HCV replication as has been suggested in the literature. This does not mean that the subtypes and quasispecies of HCV RNA does not exist *in vivo*, in fact they do. But that has little to do with the biology of infectious, replicating HCV virions. Although one could debate as to which cell types are critical for inducing maladies in patients, that point will remain debatable and vary with the point of view of the investigator. HIV-1 and Epstein-Barr virus (EBV) are excellent examples to illustrate this point. Since the discovery of HIV, many researchers were certain that CD4+ T cells were the only cells susceptible to HIV-1 infection. The discovery that monocytes/macrophages were not only infectable, but also acted as a reservoir entirely changed the discourse. Similarly, it was discovered that EBV infects not only B-cells, but also the epithelial cells of the nasopharynx, which serves as a reservoir of the virus.

Data presented here shows that our system can culture HCV that does not differ from the ones found in patients' blood. Culturing the virus for extended periods of time appears to produce only minor changes in the 5'UTR. However, culturing the HCV in different cell types did cause detectable changes. The two cell types that produced the most notable effects are T-cells (112A), which caused a loss of major variants and consequent increase in entropy, and non-committed lymphoid cells, which caused an unusual shift from a T to a C at position 106. Both of these cell types also infrequently caused an insertion of an extra C in a string of C's in the 5'UTR. What remains to be studied carefully are the effects of HCV infection on cellular gene expression. We strongly feel that HCV is a slowly replicating virus, which explains both the low titer of infectious virus in patients and in cell culture and the long incubation period before the development of an acute disease. The high titer of viral RNA in plasma/serum is not the same thing as infectious virus. Similarly, in the case of HTLV-1 and HTLV-2, the virus production in the plasma/sera of patients remains low [41]. Despite best efforts, *in vitro *production of HTLV-1 and HTLV-2 to date remains low as well [42]. In addition to the slow nature of HCV replication, we did not observe any subtype or quasispecies in either the primary isolate from the patients or CIMM-HCV. Our system is, therefore, well suited for studying the biology and immunology of HCV infections. It is also an excellent system for testing the efficacy of candidate drugs and advancing ideas for vaccines.

## Methods

### *In vitro *culture system

Our system is a two-step procedure: (1) infection of macrophages with HCV derived from patients' blood (2) infection of freshly transformed B-cells obtained from human fetal cord blood with HCV obtained from the macrophages. Three types of samples were analyzed: (1) HCV found in serum or plasma of patients (2) HCV produced by macrophages, designated the primary isolate, and (3) the HCV produced by B-cells after varying periods of time. Each patient was given a unique ID, and the isolates from that patient used the same ID with added qualifications such as the primary isolate (i). Each subsequent transfer into fresh uninfected cells was given a designation of T1, T2, up to T7. CIMM-HCV produced by B-cells infected a variety of other cell types, including human neuronal precursors [2].

### RT-PCR

RNA was purified and a nested RT-PCR performed as described in Revie, *et al*. [2] using Taq polymerase (Promega) and Fidelitaq (US Biochemicals).

### Sequencing

PCR product from the nested PCR was cloned using Invitrogen's ZeroBlunt or TopoTA cloning kits. Plasmid DNA from the clones was amplified using Amersham's Templiphi. The DNA was sequenced using a Beckman CEQ8000 Genetic Analysis system. A 269 bp fragment that spans most of the 5'UTR was sequenced for a minimum of 25 clones of each sample. In order to ensure high quality analyses, only clones that had identical sequences for both strands were analyzed. All methods followed the manufacturers' protocols.

### Bioinformatics

The set of sequences for a particular sample was aligned and analyzed using Lasergene's DNASTAR. Although a 50% identity is commonly used for calling a consensus, we used a cutoff of 60% [43]. After a consensus sequence was obtained, each sequence from the clones was compared to the consensus sequence in order to determine the variability of each sample. The numbers for the base positions that are reported here are the bases compared to the positions of the full length genome of HCV-N [44]. Control HCV sequences for strains 1a and 1b were obtained from the HCV sequence database [24].

Complexity of the variation was calculated as Shannon entropy and Pn complexity described by Cabot *et al*. [19] and Pawlotsky *et al*. [20]. Normalized Shannon entropy measures the proportion of different viral genomes in a sample, while Pn measures the proportion of polymorphic sites within a population. Shannon entropy is a measure of the number of different genomes present in the sample, and Pn values measure sequence variability.

### Accession numbers of HCV sequences used for genotyping

The 5' UTR sequences have the GenBank accession numbers EF028177 to EF028185, EF028187, and EF028190 to EF028193.

## Declaration of competing interests

All intellectual rights are reserved by the California Institute of Molecular Medicine (CIMM), and all aspects of this work were performed by CIMM. There are no competing interests between California Lutheran University or any other body and CIMM.

## Authors' contributions

SZS performed the biological work and the isolations, transmissions, and retransmissions of HCV. JGP performed the clinical work, recruitment of patients, and procurement of specimens. DR, MOA, RSB, DB, and NC performed the molecular work.
